# Mediation analysis of relationships between chronic inflammation and quality of life in older adults

**DOI:** 10.1186/s12955-016-0452-4

**Published:** 2016-03-22

**Authors:** Alexandra C. H. Nowakowski, Katelyn Y. Graves, J. E. Sumerau

**Affiliations:** Department of Behavioral Sciences and Social Medicine, College of Medicine, Florida State University, 1115 West Call Street, Suite 3200, Tallahassee, FL 32306-4300 USA; Department of Sociology, College of Social Sciences and Public Policy, Florida State University, Tallahassee, FL 32306 USA; Department of Sociology, College of Social Sciences, Mathematics, and Education, University of Tampa, Tampa, FL 33606 USA

**Keywords:** Inflammation, Quality of life, Elaboration modeling, NSHAP, Logistic regression

## Abstract

**Background:**

This article summarizes exploratory analyses of relationships between chronic inflammation, its physical consequences, and quality of life (QoL). It summarizes key findings from preliminary analyses, and contextualizes these results with extant sociomedical literature to recommend directions for future research.

**Methods:**

Cross-sectional data from the National Social Life, Health, and Aging Project (NSHAP) were used to explore these relationships. Inflammation was assessed via the biomarker C-reactive protein (CRP). We examined associations between CRP levels and two different domains of QoL: happiness with life in general and happiness with intimate relationships. We used ordinal logistic regression with companion OLS models and Sobel-Goodman tests to assess potential mediation, and also conducted a variety of sensitivity analyses.

**Results:**

Findings suggest that mediation pathways for the overall association between chronic inflammation and QoL may differ markedly across particular outcome constructs. Specifically, it shows mediation potential for the clinical sequelae of chronic inflammation in frameworks using happiness as an outcome measure, but not in those using relationship satisfaction. Disability appears to mediate the effect of inflammation by 27 %; chronic pain appears to exert a similar mediation effect of 21 %.

**Conclusions:**

Pain and disability linked to chronic inflammation appear to play a small but significant mediating role in the overall reduction in QoL observed among older adults with biomarker evidence of chronic inflammation. We note that these patterns are best framed as dynamic elements of a complex causal fabric, rather than powerful determinants that override other factors contributing to QoL. Hypotheses for further exploration using longitudinal data from the NSHAP are thus offered, pending availability of Wave III data in future years.

## Background

The relationship between chronic conditions and quality of life (QoL) represents a key focus in medical sociology research [1]. Many of the conditions that sociomedical scientists have explored are inflammatory in nature [2]. Examples include arthritis [3–8]; inflammatory bowel disease [9–14]; interstitial cystitis [15–19]; and asthma [20–22]. These studies consistently show that chronic disease can reduce QoL in a variety of domains [23]. Consistent negative associations have been identified between chronic inflammatory disease and overall happiness with life, as well as satisfaction with intimate relationships [24].

Literature on inflammatory disease and QoL suggests that these negative associations may be mediated by common medical consequences of chronic illness [25]. These sequelae generally include functional limitation and/or persistent pain [2]. Sociology distinguishes between “limitation” and “disability” as well as “pain” and “suffering” by illuminating how physical experiences become sources of social disadvantage [26]. In turn, these challenges can reduce QoL by producing feelings of social alienation [27], derailed progress [28], and lost identity [29]. These changes may arise via multiple social and clinical mechanisms associated with chronic disease, including the pathology of inflammation and any resultant clinical diagnoses.

Biomarker data can offer insight into the potential mediating impact of formal diagnosis for people who live with inflammatory pathology. Indeed, medical sociologists have begun to use biomarker data as a complement to diagnosed inflammatory disease in assessing health. The common inflammatory biomarker C-reactive protein (CRP) often captures chronic inflammation particularly well due to its unique attributes [30]. Consequently, it has emerged as a predictor of interest in many sociological studies of inflammatory disease. Reliable associations have been found between blood levels of CRP and a variety of QoL outcomes [24, 31].

Much of the sociological literature on relationships between chronic disease and QoL focuses on how these conditions produce consequences that are both medical and social in nature. Indeed, a key contribution of this literature is the idea that disease only becomes illness under certain circumstances: when it affects identity formation processes [26] and/or threatens individuals’ sense of being whole [32]. These same concepts offer insight into how common sociomedical sequelae from chronic conditions may mediate associations between inflammatory chronic disease and QoL. Specifically, relationships between inflammatory biomarker levels and QoL may be mediated by diagnosis, disability, and pain.

Diagnosis reflects a variety of physical experiences that can later translate to disadvantages in the social world. Indeed, receiving a diagnosis formalizes a given person’s transition into the role of *patient* [26]. This shift occurs through the process of medicalization [33] because health care providers assert social control over people entering into the patient role [34] and thus confer legitimacy [35–37]. Diagnosis can facilitate access to roles [38], accommodations [39], resiliency [40], coherence [41, 42], and relationships [43] that help people cope. At the same time, diagnosis can also brand people as deviant [36, 37], alienate people from themselves [41, 42], and instill feelings of hopelessness [28, 41]. Diagnosis also brings expectations to work actively toward recovery [38], remain positive and hopeful [39], and weather suffering with good humor [42]. For people with chronic conditions, these expectations are often unrealistic [39] and the resulting disappointment can lead others to offer less support over time [41].

Like diagnosis, disability is as much a social construct as it is a medical one [26]. Functional impairment becomes *disability* when it becomes inhibiting in social contexts [32]. This can bring a variety of negativeconsequences for social life [44]. Coping with these consequences can also lead to unexpected positive experiences. Having a physical disability thus does not necessarily result in low QoL, but can introduce significant impediments to people’s overall sense of well-being and fulfillment [26]. These challenges can include stigma [37, 45, 46], sanctions for not responding appropriately to the discrediting behavior of others [42, 45, 46], and embarrassment from inability to meet normative expectations of behavior [41, 47].

Pain similarly becomes *suffering* when it produces adverse social consequences [26]. Pain itself can prompt people to seek medical care [26] and receive a diagnosis [39], and thus afford access to some of the benefits of this process. Yet by the same token, both the receipt of a diagnosis and the experience of pain can levy dramatic negative consequences on social life., Showing evidence of pain can negatively impact both social [26] and medical [39] interactions by introducing feelings of burden and guilt [26], as well as frustration and disappointment [39] that eventually lead to anger and [26] withdrawal of social support [41]. Likewise, medical providers frequently express disappointment in people who have not met the standard demands of the sick role [39]. Specifically, the sick role involves the core expectation that the person occupying it will eventually recover and be free of symptoms. Providers often demonstrate not only disappointment, but also frustration and even outright anger with people who continue to experience pain from a chronic condition despite actively undergoing treatment [26].

Sociomedical sequelae of chronic disease thus constitute generic processes [48] that entrench inequality in health-related quality of life [49]. Although relatively few sociologists have studied these dynamics for chronic inflammation and related diseases specifically, these conditions share many clinical and social features with other persistent diseases [23, 50]. Sociologists of health can thus use data on chronic inflammatory pathology and conditions to explore how pain, disability, and diagnosis may mediate the overall experience of disease. Many of the datasets that allow for exploration of these relationships are relatively new, and thus offer only limited insight into longitudinal processes. As additional waves of data are collected for major biosocial projects, cross-sectional inquiry can establish a foundation for more sophisticated studies using panel designs when sufficient data become available. We thus used the two available waves of data from a relatively new biosocial dataset on older United States adults to test the mediational effect of inflammatory biomarkers linking chronic diseases and QoL. In this manuscript we build on our team’s prior work linking inflammatory biomarker levels and key elements of QoL, as well as social disadvantage and chronic inflammation.

## Methods

We used data from Waves I and II of the NSHAP. This biosocial dataset provides information on physical, mental, and social health among United States residents aged 57 to 85 at Wave I. The dataset includes 3005 individual cases in total. NSHAP data documentation describes the study sample as “a nationally representative probability sample of community-dwelling individuals” [51]. African Americans, Latinos, males, and persons 75 to 85 years of age are oversampled to boost statistical power [51]. Data are collected via a combination of questionnaires, in-home interviews, and clinical exams (Table 1).

**Table 1:** Characteristics of sample population from NSHAP wave I (*n* = 1684)

Construct	Variable	Units	Mean	SD	Range
Quality of life	Generally feeling happy	Points	3.61	0.86	1–5
	Happiness with relationships	Points	5.91	1.61	1–7
Chronic inflammation	C-reactive protein serolevel	mg/L	1.72	1.46	0–5.98
Medical sequelae	Functional disability	Points	1.10	1.75	0–9
	Pain while walking	Yes/No	0.38	0.49	0–1
	Diagnosed chronic condition	Yes/No	0.88	0.32	0–1
Chronological age	Years of age	Years	69.6	7.90	57–85
Sex identity	Male	Yes/No	0.50	0.50	0–1
	Female		0.50	0.50	
Racial background	White	Yes/No	0.75	0.43	0–1
	Minority		0.25	0.43	
Educational attainment	Years of education	Years	12.6	4.08	0–32

The NSHAP is designed as a panel dataset with multiple repeated measures. However, only two waves of NSHAP data are currently available, making the data essentially cross-sectional at present for research questions requiring three or more waves of data for rigorous assessment in longitudinal context. However, the older age of the study population [52] does allow for conjecture about long-term impacts on QoL from a variety of chronic health conditions. A substantial foundation also exists [53] for using cross-sectional data to generate hypotheses about mediation processes. We thus used available data from the NSHAP to begin exploring these processes, with the goal of recommending trajectories for future research. We used Wave I data to compute our final models because we did not have three waves of data to represent each timepoint in the mediation pathway. This strategy offered the additional advantage of facilitating comparison of our results in this study to prior work by our team. However, we did conduct a variety of sensitivity analyses using the Wave II data, which we describe at a later point in the manuscript.

To assess inflammation we used serolevels of CRP, a substance that circulates in the blood of individuals experiencing significant amounts of inflammation. We note that CRP is a biomarker of inflammation rather than a direct measure of inflammation itself. CRP provides compelling evidence that inflammation is present. The NSHAP contains a continuous measure of total serum CRP. We retained over 87 % of total participants with real data on the CRP variable, excluding cases with very high values due to concerns about biologic plausibility [49]. According to NSHAP data documentation, these extremely high values for the approximately 300 additional study participants whose data were not used likely owed to measurement error [51]. Our initial analytic sample contained 1684 people whose observed CRP serolevels varied between 0 and 5.98 milligrams per liter [24].

Prior to running any regressions, we made three sets of comparisons to ensure that using only data on participants with a successful CRP assay and then further restricting the range of CRP values used did not introduce differential bias. First, we compared the full sample of NSHAP participants to just those with real data for the CRP variable. Second, we compared those respondents with a successfully assayed CRP value to those without one. Third, we compared the participants with real CRP values who were excluded due to concerns about biologic plausibility to those whose values were included. These sensitivity analyses helped us avoid excluding populations that would yield substantive additional insight into our research questions.

We measured key elements of QoL using both emotional and relational constructs to facilitate comparison of results across domains. The NSHAP’s “generally feeling happy” and “overall happiness with relationships” variables, provided data for each QoL component outcome respectively. These constructs were assessed using validated questions similar to those from the NSHAP’s immediate predecessor and foundational inspiration, the National Health and Social Life Survey [51]. These questions were set up as Likert scales that included numeric levels with corresponding text descriptors for the endpoints of each scale. Questions were structured as follows:

***“If you were to consider your life in general these days, how happy or unhappy would you say you are, on the whole?”***

- ***1 – unhappy usually***
- ***2 – unhappy sometimes***
- ***3 – pretty happy***
- ***4 – very happy***
- ***5 – extremely happy***

***“Taking all things together, how would you describe your relationship with your partner on a scale from 1 to 7 with 1 being very unhappy and 7 being very happy?”***

- ***1 – very unhappy***
- ***2***
- ***3***
- ***4***
- ***5***
- ***6***
- ***7 – very happy***

Both outcomes were preserved as ordinal measures rather than dichotomized. Initially we also explored subcomponents of these overall outcomes, which the NSHAP captures as separate variables such as “physical satisfaction with relationships” and “emotional satisfaction with relationships”. However, we found that analyzing these component outcomes separately afforded little insight in excess of that yielded by the NSHAP’s more general questions about happiness and relationship satisfaction.

The “generally feeling happy” variable is an experiential measure of subjective well-being that assesses how frequently respondents have experienced happiness in the 12 months prior to participating in interviews. The “overall happiness with relationships” variable captures feelings of happiness specifically with respect to intimate partnerships in the same time window. Because having a partner can itself contribute to subjective well-being, we also did some preliminary analysis prior to running regressions that revealed nearly all of the people in our sample were either married or cohabitating without being married. Incorporating partnerships as a control variable thus added little value.

We operationalized physical disability by aggregating information about activities of daily living and instrumental activities of daily living with which study participants experienced difficulty. We chose these measures because they are both highly validated in the literature [44] and corroborated by NSHAP interviewers after participants’ initial reporting [51]. We added binary scores for each of the seven activities of daily living (walking one block, walking across a room, dressing, bathing, eating, getting in and out of bed, toileting) and two instrumental activities of daily living (driving in daylight, driving at night) assessed by the NSHAP. Specifically, we aggregated each of the component ADL variables from the NSHAP into a summative index by adding up possible values (0 or 1 in all cases) for a maximum disability score of 9 and a minimum score of 0, depending on how many activities people reported experiencing difficulty in completing. We used this index to model the potential mediating impact of functional impairment in associations between chronic inflammation and QoL.

We measured persistent pain using the NSHAP’s variable capturing whether or not people experience pain while walking, which had real values for most study participants and offered a reasonable proxy for chronic pain across a variety of common daily activities. The NSHAP also contains two additional variables that capture pain during other activities: sexual intercourse and getting up from a sitting position. Our preliminary examination of the NSHAP data, including exploratory factor analysis of each measure’s unique value for modeling QoL constructs, suggested that “pain while walking” offered the best insight into our outcomes of interest. In addition, these other measures of pain were not available for a large portion of cases. The binary “pain while walking” variable thus provided the best means of modeling the potential mediating impact of persistent pain on relationships between inflammation and QoL.

We operationalized diagnosis status via a single variable in the NSHAP that captures whether or not people have ever been diagnosed with a chronic condition. The dataset also captures information about 18 specific types of chronic conditions, including three that may include inflammatory pathology (asthma, gastrointestinal disease, and arthritis). However, we did not use these variables for two specific reasons. First, they only capture a small subset of the complete spectrum of health conditions that involve chronic inflammation. Second, they may capture conditions that are not inflammatory in nature, such as gastric ulceration or bowel perforation. The binary “any chronic condition” variable thus provided an ideal means of exploring the general mediation potential of a clinical diagnosis.

We used logistic regression to explore possible mediation relationships between chronic inflammation, its common medical sequelae, and two different types of QoL. For each QoL outcome, we first computed bivariate ordinal logistic models of the relationships between inflammation and QoL. We also generated bivariate ordinal and binary logistic models of associations between inflammation and each of its common sequelae, as well as bivariate ordinal logistic models using each suspected mediator to predict each QoL outcome. Next, we incorporated each of the three potential mediators into bivariate ordinal models of inflammation and QoL. For each outcome variable we computed trivariate models incorporating suspected mediators in isolation, as well as more complex models using all three sequelae as an aggregated block.

We assessed potential effect mediation using Sobel-Goodman tests on a series of companion models computed using OLS regression. Although allowed by some statistical software packages including the one we used, performing these tests on ordinal logistic models is widely viewed to be theoretically inappropriate. Performing these procedures instead on the companion OLS models we computed, which yielded substantively identical results to our original ordinal logistic ones, allowed us to examine the plausibility of a mediation effect from common medical sequelae of chronic inflammation, as well as the apparent proportion of the predictor’s total effects mediated by the intervening variables.

To guard against reporting results that were merely artifacts of our specific modeling framework and testing strategy, we also performed a series of sensitivity analyses. First, we computed regressions for all hypothesized models using structural equation modeling with bootstrap standard errors and compared results to those obtained from ordinal logistic and OLS models. Comparing results across these different modeling frameworks allowed us to achieve reasonable confidence in the findings from our ordinal logistic regressions and companion OLS models. Second, we compared our results from purely cross-sectional modeling to those obtained with a variety of different limited-longitudinal modeling specifications, finding results to be substantively equivalent. Specifically, we used CRP serolevels from Wave I; measures of disease sequelae at Waves I and II as appropriate; and measures of QoL components at Wave II. This allowed us to compute longitudinal bivariate models for each component pathway in the overall mediation analysis, and limited-longitudinal expanded models for the full framework, to which we then compared our models using only Wave I data.

Finally, we computed a set of models incorporating information about a variety of other factors that can lead to social disadvantage. Specifically, we included data on sampled NSHAP participants’ age in years, sex identity, ethnoracial background, and educational attainment in years. While the idea of “controlling” for such factors is more abstract than concrete, including information about social status and position can afford meaningful insight about potential confounding of overall associations between inflammation and QoL. Moreover, incorporating structural covariates can illuminate possible fundamental causation of chronic inflammation by social disadvantage, a process in which medical sociologists have increasingly taken interest [49, 54, 55]. We present models that frame social structural disadvantage as a fundamental cause of chronic inflammation because the literature increasingly supports this perspective.

For all ordinal logistic models, we performed Brant tests to ensure that parallel regression assumptions were fully met. No models yielded significant results on these tests, indicating that all models satisfied the basic assumption that the odds of moving between any two successive contiguous categories were roughly equivalent across all levels of each outcome. Diagnostic tests were not performed on binary logistic models because parallel regression is not an issue for outcome variables with only two possible values.

After computing preliminary models for every analyzed pathway, we ran sensitivity analyses incorporating data completeness elements and survey design variables from the NSHAP. This allowed us to account for the potential influences of participant non-response and data clustering. We ran models incorporating the survey weighting variables and compared results with those obtained from our initial regressions. To assess the potential impact of missing data, we also ran a series of sensitivity analyses using complete case samples (versus the small variations in sample sizes that appeared with each bivariate model in the hypothesized mediation pathway). Because these analyses either left our results substantively unchanged or slightly improved the significance of parameter estimates, we present unadjusted results for ease of interpretation.

This research study was performed in accordance with the principles for protecting data on human participants outlined in the Declaration of Helsinki. It was approved by the Florida State University Human Subjects Committee on June 23, 2014 with reference number 2014.12860. This approval was renewed on March 31, 2015 with reference number 2015.15273. Our study used only secondary data obtained from Waves I and II of the NSHAP. No active data collection with human participants was conducted at any point during our research process. Consequently, we have no consent documents of our own to share. All uses of data for this research fell within the scope of accepted activity for approved NSHAP data users. No individual NSHAP participant data are reported in this manuscript.

## Results

We began by assessing the ability of inflammatory biomarker levels to predict each of the suspected mediating constructs (i.e., disability, pain, and diagnosis). Table 2 shows results from bivariate logistic regressions of each hypothesized mediator on CRP serolevels. In developing each model, we computed odds ratios for the relevant indicator. As Table 2 shows, we found reliable preliminary evidence to suggest pain, disability, and diagnosis could serve as mediators of the overall association between chronic inflammation and QoL. Specifically, CRP serolevels demonstrated significant ability to predict at least one indicator of each variable. These results were observed in both raw examination of CRP parameter significance attenuation by the hypothesized mediators, and Sobel-Goodman testing (via companion models) of the purported mediation effects.

**Table 2:** Sociomedical sequelae regressed on C-reactive protein

Outcome	Predictor	Odds ratio	Model *χ* ^2^	Pseudo R^2^
Functional disability	C-reactive protein serolevel	1.18*** (1.11, 1.26)	25.62***	0.0060
Pain while walking		1.17*** (1.09, 1.26)	18.61***	0.0098
Diagnosed chronic condition		1.13* (1.01, 1.26)	4.85*	0.0040

† *p* < 0.10, * *p* < 0.05, ** *p* < 0.01, *** *p* < 0.001

95 % confidence intervals shown in parentheses

For disability, we found that each additional milligram per liter of CRP was associated with 18 % greater odds of experiencing high levels of functional impairment. This association is significant with a *p*-value less than 0.001. For pain, we found that each additional milligram per liter of CRP was associated with 17 % greater odds of experiencing chronic pain while walking. This association is also significant with a *p*-value less than 0.001. For diagnosis, we found that each additional milligram per liter of CRP was associated with 18 % greater odds of being diagnosed with any chronic condition. Once again, this association is significant with a *p*-value less than 0.001.

Next, we examined the potential ability of these sociomedical variables to mediate overall associations between inflammatory biomarkers and QoL outcomes. We did so by incorporating each of the sociomedical indicators into models of QoL outcomes regressed on CRP serolevels. This allowed us to assess the degree to which sociomedical sequelae of chronic inflammation attenuated the significance of CRP itself in predicting each QoL indicator. Table 3 summarizes results from these analyses. Each of the three types of sociomedical sequelae (i.e., disability, pain, diagnosis) attenuated the overall predictive value of CRP for QoL outcomes associated with inflammatory biomarker levels.

**Table 3:** Potential mediation effects of individual sociomedical sequelae

Outcome	CRP serolevel	Mediator	Parameter estimate	Proportion of effect mediated	Sample size
Generally feeling happy	0.93* (0.87, 0.99)	Functional disability	0.84*** (0.80, 0.89)	0.27***	1,421
	0.91** (0.85, 0.98)	Pain while walking	0.56** (0.46, 0.69)	0.21***	
	0.91** (0.85, 0.96)	Diagnosed chronic condition	0.65** (0.50, 0.86)	0.05	
Happiness with relationships	0.91** (0.85, 0.98)	Functional disability	0.98 (0.93, 1.04)	0.00	1,376
	0.94† (0.88, 1.00)	Pain while walking	0.88 (0.71, 1.08)	0.07	
	0.92** (0.86, 0.97)	Diagnosed chronic condition	0.97 (0.73, 1.29)	0.01*	

† *p* < 0.10, * *p* < 0.05, ** *p* < 0.01, *** *p* < 0.001

95 % confidence intervals shown in parentheses

We also assessed component fit for each covariate as well as whether or not these variables improved the overall fit for each QoL outcome. We did so by computing Bayes Information Criteria for initial models (e.g., regressions of QoL outcomes on CRP levels using only cases with real values on the included covariate) and final models (e.g., regressions of QoL outcomes on CRP levels and sociomedical sequelae). Each of the three sociomedical sequelae significantly predicted by inflammatory biomarker levels (disability, pain, diagnosis) substantially improved the overall fit of models for general happiness, but not for relationship happiness. Modeling for the happiness outcome was consistently and significantly improved by incorporation of all three sociomedical sequelae, whereas overall fit for regressions of the other outcome became worse. These results mirrored our findings from Sobel-Goodman testing on companion models of single mediators in the pathway between CRP and each form of QoL.

We then computed models for each QoL outcome incorporating the three common sequelae of chronic inflammation as a block. Findings from integrated modeling of all three variables support the idea that these constructs may collectively mediate the relationship between chronic inflammation and QoL, but more in some domains than in others. Incorporating sociomedical sequelae of chronic inflammation as a block reveals strong mediation potential for a measure of overall happiness, but virtually none for a measure of happiness with intimate relationships. Wald chi-square testing revealed that adding the block of inflammation consequences substantially depreciated model fit, as did overall model likelihood testing for the expanded regression equation. By contrast, fit tests for the expanded model regressing overall happiness on CRP and the potential sequelae of inflammation suggested superior predictive ability for the more complex model.

Results from these analyses are shown in Table 4. When happiness with life in general is modeled as the outcome, two of the three common sequelae of inflammation appear to play a mediating role. Disability appears to exert the strongest mediating influence, followed by pain while walking. In fact, when modeled together with pain and disability, diagnostic status does not appear to exert an independent mediating influence. Diagnosis with any chronic condition, however, is often highly collinear with both pain and disability, which suggests this finding does not necessarily mean that diagnosis does not independently influence the relationship. Indeed, sensitivity analyses reveal a consistent relationship between diagnosis with one or more chronic conditions and decreased happiness with life.

**Table 4:** Possible collective mediation effects from sociomedical sequelae variables

Outcome	Variable	Model 1	Model 2	Sample size
Generally feeling happy	C-reactive protein serolevel	0.90** (0.85, 0.96)	0.93* (0.87, 0.99)	1,288
	Functional disability	-	0.86*** (0.80, 0.91)	
	Pain while walking	-	0.70** (0.56, 0.87)	
	Diagnosed chronic condition	-	0.85 (0.62, 1.17)	
	*Prob > χ* ^*2*^	0.0028	0.0000	
	*Pseudo R* ^*2*^	0.0028	0.0181	
Overall happiness with relationships	C-reactive protein serolevel	0.92* (0.86, 0.98)	0.92* (0.85, 0.99)	1,255
	Functional disability	-	0.99 (0.93, 1.05)	
	Pain while walking	-	0.92 (0.72, 1.15)	
	Diagnosed chronic condition	-	1.17 (0.85, 1.62)	
	*Prob > χ* ^*2*^	0.0264	0.1634	
	*Pseudo R* ^*2*^	0.0014	0.0019	

† *p* < 0.10, * *p* < 0.05, ** *p* < 0.01, *** *p* < 0.001

95 % confidence intervals shown in parentheses

## Discussion

Our findings provide support for hypothesized relationships between chronic inflammation, pain, disability, diagnosis, and QoL. Specifically, our results reveal that pain, disability, and diagnosis may individually and collectively mediate associations between biologically-demonstrated chronic inflammation and QoL. These findings are consistent with evidence suggesting relevant sequelae of chronic disease and the ways such constructs may mediate relationships between health status and QoL.

As suggested previously, research on how pain and disability impact QoL yields more consistent evidence concerning the direction of influence. Specifically, sociologists have found that functional impairment and chronic pain generally reduce QoL in multiple domains [26]. Clinical scientists have observed similar patterns among people with many specific inflammatory conditions [23]. Considering that sociologists explain these declines in QoL via changes in health that threaten the integrity of people’s relationships with self and others [32], such findings reveal equal relevance for sociomedical sequelae from specific chronic conditions and consequences associated with broader experience of chronic disease.

Our findings also support evidence concerning mediating impacts of diagnosis on the association between health status and QoL. On the one hand, diagnosis with any chronic condition is typically associated with at least modest declines in QoL [56], but on the other, diagnosis can confer important resources and support systems [57]. The fact that diagnosis is not as strong or consistent a mediator between chronic inflammation and QoL may thus indicate that this variable exerts influence through multiple pathways. People whose conditions have been diagnosed may experience unhappiness and grief, but may also experience relief from anxiety that stems from reduced uncertainty about health and greater ease communicating their illness experience [41]. Because we measured only diagnosis with any chronic condition as opposed to specific diseases, findings may also reflect that not all chronic conditions influence QoL in the same ways.

Our findings for both the pain and disability variables also illuminate relevant deficiencies of the sick role concept as they relate to persistent inflammation. Non-visible sequelae from inflammatory disease, such as chronic pain, may cause the sympathy of friends and loved ones to wane at greater speed [26]. This phenomenon is likely mediated by the discrediting role of invisible illness, which lacks social legitimacy [37]. Functional disability presents a different set of concerns, especially when the impairment can be seen by others. People who experience disability from chronic disease may experience relative declines in social isolation if their impairment becomes severe enough to demand outside caregiving [58]. However, gains in social support mediated by caregiving often occur outside the realm of intimate partnerships [59]. In cases where partners do become primary caregivers, partners experience high risk of morbidity due to the role conflict and captivity that can often result from providing care for an ailing partner [58].

Taken together, these findings suggest that the mechanisms by which the common sociomedical sequelae of chronic inflammation may mediate the overall relationship between inflammatory disease and QoL are highly nuanced. Likewise, these mechanisms are often dynamic and multifaceted in terms of directional influence: A given medical consequence of chronic inflammation may exert important positive and negative influences on QoL synergistically. We therefore encourage caution in interpreting findings concerning the ability of these constructs to improve the overall fit of models relating chronic inflammation to QoL. Non-significant improvements in fit do not suggest that a given sociomedical sequelae variable confers no predictive value, but rather that decomposing this variable into smaller elements may be necessary to assess the different impacts it exerts. We also note that happiness itself is a nuanced concept, both within and outside of intimate relationships. Our reviewers for the first draft of this manuscript pointed out that experiential measures of happiness both offer valuable insight and pose key limitations.

Indeed, we further urge caution interpreting our findings due to several limitations in our measurements. First, as noted above the two outcome measures are experiential and reflect a relatively large time window of 12 months. As such, they do not necessarily reflect variations in daily experience or the complexity of happiness as an emotion. Second, we also did not have access to a complex overall measure of chronic pain. While the “pain while walking” variable offered a reasonable proxy given the community-dwelling and age-diverse makeup of the NSHAP population, it did not capture nuances of pain or its impact on daily activities. Third, as with any biomarker, although we excluded missing measurements and those with obvious errors, there may be some mildly inaccurate estimates of participants’ CRP values in the analytic data stream. Although bias introduced by small measurement errors is likely to be to the null [30], we do note that our study mirrors other biosocial analyses in the need for continued replication and refinement of findings with varied data sources and populations. Fourth, although extant literature validates CRP as a general measure of chronic inflammation, less is known about how well CRP predicts the manifestation and severity of specific inflammatory conditions. Care should thus be taken to further refine such knowledge as biosocial research expands in coming years.

We also note an additional key limitation in the study design itself, albeit one that we cannot address at this time given lack of available data. As previously noted, we did not have three waves of data for use as only Waves I and II of NSHAP are presently available. We are thus unable to establish the full impact of mediation or provide panel design type conclusions. We certainly encourage fellow scholars to continue this research with additional waves of NSHAP as they become available, and to incorporate more robust uses of advanced longitudinal modeling techniques and SEM frameworks using appropriate software.

Despite these limitations, our study also presents some important strengths other researchers may build on in future research. First, our analyses focused on an older age study population, which grants greater probability of hypothesizing likely nuances and outcomes in the coming years. Second, we were able to demonstrate some longitudinal elements of mediation by utilizing two waves of data throughout our analyses. Further, we possessed solid biosocial data on inflammation, which is necessary for teasing out nuances and influences along biological, sociological, and psychological dimensions of health and well being. Finally, the NSHAP is built upon evidence-based inclusion and exclusion criteria, which is necessary for accurate accounts of health and illness experience within and between populations. We would thus argue that with the proper caution researchers may build upon our strengths to further understand the complexities of biosocial experiences of chronic conditions.

## Conclusions

Taken together, our findings suggest that sociomedical sequelae may indeed serve as mediators of the relationship between chronic inflammation and QoL. However, they also suggest that social pathways mediating the hypothesized relationship between chronic inflammation and QoL may differ fundamentally for emotional versus relational outcomes. More broadly, our findings indicate a need for more nuanced research on social mechanisms that may mediate the effects of chronic inflammatory disease across a variety of different dimensions of QoL. Indeed, this interpretation mirrors robust evidence from sociological literature on QoL measurement concerning the importance of capturing multiple outcomes [60]. Findings also reflect potential nuances in the impact of specific chronic conditions on QoL, and the diversity of these experiences among people with different health profiles and social locations.

Our analyses of disability, pain, and diagnosis as potential mediators of the relationship between chronic inflammation and QoL recommend three tentative conclusions. First, the overall association between chronic inflammation and QoL is likely to be mediated by sociomedical sequelae. Second, this apparent mediation is partial rather than complete. Our findings suggest that chronic inflammation generally exerts influences on QoL in excess of those explained by disability, pain, and diagnosis. Third, sociomedical sequelae appear to mediate the relationship between chronic inflammation and emotional QoL more strongly and consistently than that between inflammation and relational QoL. Our findings suggest there may be much to learn from potential mediators between chronic disease experience and varied components associated with overall QoL. We specifically recommend extending this analytic framework across a variety of chronic inflammatory conditions and associated biomarkers.

## Abbreviations

CRP: C-reactive protein
NSHAP: national social life, health, and aging project
QoL: quality of life
